# Mechanical Properties of Ultra-High Performance Concrete with Coal Gasification Coarse Slag as River Sand Replacement

**DOI:** 10.3390/ma15217552

**Published:** 2022-10-27

**Authors:** Ziqi Zhu, Xiaoqing Lian, Xiaowei Zhai, Xiaojun Li, Muhong Guan, Xiang Wang

**Affiliations:** 1 CHN Energy Shendong Coal Group Co., Ltd., Shenmu 719315, China; 2 School of Materials Science and Engineering, Xi’an University of Science and Technology, Xi’an 710054, China; 3 School of Safety Science and Engineering, Xi’an University of Science and Technology, Xi’an 710054, China; 4 Anhui Water Conservancy Development Co., Ltd., Bengbu 233000, China

**Keywords:** coal gasification slag, ultra-high performance concrete, microstructure, mechanical properties, hydration

## Abstract

Coal gasification coarse slag (CGCS) is a by-product of coal gasification. Despite its abundance, CGCS is mostly used in boiler blending, stacking, and landfill. Large-scale industrial applications of CGCS can be environment-friendly and cost saving. In this study, the application of CGCS as a substitute for river sand (RS) with different replacement ratios in ultra-high performance concrete (UHPC) was investigated. The effects of CGCS replacement ratios on the fluidity and mechanical properties of specimens were examined, and the effect mechanisms were explored on the basis of hydration products and the multi-scale (millimetre-scale and micrometre-scale) microstructure analysis obtained through X-ray diffraction (XRD), scanning electron microscopy, and X-ray energy-dispersive spectroscopy. With an increase in the CGCS replacement ratio, the water–binder ratio (w/b), flexural strength, and compressive strength decreased. Specimens containing CGCS of ≤25% can satisfy the strength requirement of non-structural UHPC, with flexure strength of 29 MPa and compressive strength of 111 MPa at day 28. According to the XRD results and multi-scale microstructure analysis, amorphous glass beads in CGCS positively influenced ettringite generation due to the pozzolanic activity. Porous carbon particles in CGCS showed strong interfacial bonding with cement slurry due to internal hydration; this bonding was conducive to improving the mechanical strength. However, CGCS hindered hydration in the later curing stage, leading to an increase in the unreacted cement and agglomeration of fly ash; in addition, at a CGCS replacement ratio of up to 50%, an apparent interfacial transition zone structure was observed, which was the main contributor to mechanical strength deterioration.

## 1. Introduction

Across China, there are 14 hundred million tons of coal resources, half of which are present in the middle–upper Yellow River basin. Several modern coal chemical projects have been developed in this region [1]. The coordinated development of coal industry and environmental protection are essential for ensuring the protection and development of the Yellow River Basin. Coal gasification is one of the cleanest technologies for indirect coal liquefaction [2], and coal gasification slag is the liquefaction by-product that accounts for 15–20% of raw materials [3]. In 2020, in Yulin City, Shannxi Province, China, the annual output of CGS was approximately 3.23 million tons, but only approximately 80,000 tons (3%) of it was utilised, which is considerably lower than the national standard of the comprehensive utilisation rate (>75%) [4]. CGS is mainly used in boiler blending, stacking, and landfill. However, long-term stacking of CGS occupies a large area of land and pollutes the atmosphere, water, and soil, resulting in great environmental risks, serious waste of resources, and economic pressure. Therefore, using CGS in large-scale industrial processes is essential.

CGS can be divided into coal gasification fine slag (CGFS) and coal gasification coarse slag (CGCS). Most contemporary studies have focused on the utilisation of CGFS, which is carried by the coarse gas to the top of the gasifier furnace; CGFS comprises approximately 20% of CGS, and it has a high carbon content (between 20% and 40%) and fine particle size (66% in mass was <0.074 mm) [5]. CGFS is used as a raw material for cement; as a supplement in combustion fuels [6]; as an additive in wall materials [7]; as solid amendments [8]; and as some high-value utilisation products such as active carbon [9], sodium silicate [10], ceramic powders [11,12], and carbon–silicon composites [13]. However, CGCS, which is discharged at the bottom of the gasifier and accounts for approximately 80% of the total CGS, has been rarely investigated. CGCS has a low residual carbon content [14], because of which it is mainly used in building materials such as unburned wall materials [15], concrete [16,17,18], road pavement base/sub-base materials [19], and other cement-based materials [20,21]. Li et al. [22] studied the reaction mechanism of CGS and cement; compared with CGFS, the abundant active mineral phases in the CGCS facilitated the cementitious reaction and enhanced the mortar strength, which is consistent with the results of Liu et al. [17]. According to a study by Blaisi et al. [23], the compressive strength of mortars decreased rapidly when the replacement ratio of CGCS was up to 20%. Luo et al. [21] revealed that decarbonised CGFS resulted in higher pozzolanic reactivity than decarbonised CGCS, and the corresponding cement mortar had higher compressive strength. In the aforementioned studies, CGCS was mostly used as a cement replacement, which limited the comprehensive utilisation rate of CGCS. Limited studies have focused on the utilisation of CGCS in building materials as a fine aggregate substitute, which has a considerably higher proportion than cement in building materials. Lei et al. [19] explored the application of CGCS in semi-rigid base materials as a replacement of limestone rubble, and several optimal mix proportions were determined with 20–25% CGCS. Thus, developing a new approach for using CGCS in large-scale industrial applications as fine aggregate substitutes of building materials is necessary.

Owing to the closed packing design of mixtures and low water–binder ratio (w/b), ultra-high performance concrete (UHPC) exhibits excellent mechanical properties and durability compared with high-strength concrete (HSC) [24,25]. The preparation technology of UPHC is relatively mature; however, it is expensive and not environmentally friendly, which limits its widespread use in road and bridge materials. Quartz sands, used in mining, crushing, and grinding, are expensive, energy intensive and not environmentally friendly [23]. River sand (RS) can be easily used as fine aggregates; however, RS is a non-renewable resource, and its over-exploitation adversely affects the environment. Globally, approximately 32–50 billion tons of sand and gravel is consumed annually [26]. China is the world’s largest consumer of sand and gravel, and the total consumption in 2020 in the country was >17.8 billion tons. The conflict between the supply and demand of sand and gravel has led to a sharp increase in its cost. Thus, investigating fine aggregate substitutes of RS for use in UHPC is significant from the environmental and economic perspective.

Compared with HSC [27], UHPC requires fine aggregate substitutes with good characteristics in terms of water absorption, strength, and environmental performance. Typical substitutes used in UPHC include waste-based materials and industry by-products. Different ratios of the cathode ray tube funnel glass, that is, waste glass, were used to substitute RS in UHPC [28,29]. The addition of cathode ray tube funnel glass increased the fluidity and decreased the compressive and flexural strengths of UHPC. Jiao et al. [30] introduced waste glass sands in UHPC and observed that the fluidity and compressive strengths were significantly enhanced. Compared with UPHC with RS, for a replacement ratio of 75% (cathode ray tube funnel glass in UPHC), the compressive strength increased by approximately 30%. Yang et al. [31] observed an increase of 9.5% in the compressive strength when recycled rock dust fully substituted quartz sand in UHPC. The strength of UHPC at day 28 remained comparable to that of the UHPC with RS with an iron tailing replacement ratio up to 20% [32]. Zhu et al. [33] observed that the strength of UHPC was the highest when 60% of silica sand was substituted with iron tailings. With quartz-based mine tailings to replace quartz sand by 30% (mass fraction), the UHPC strength was comparable to that of UHPC with 100% quartz sand [34]. The compressive strength of UHPC in which 80% of quartz sand was substituted with gold mine tailings was comparable to, or higher than, that of the UHPC with 100% quartz sand [24]. Calcined bauxite aggregates were stronger than normal aggregates and exhibited a porous structure, resulting in better mechanical strength and shrinkage of UHPC [35]. However, only a few studies have investigated the utilisation of CGCS in UHPC as a fine aggregate substitute. The maximum particle size of CGCS is 2–5 mm [35], which is similar to that of RS used in UHPC without coarse aggregates; because of this, it can be used directly without mining or grinding. Moreover, most heavy metals introduced by reactants were solidified to form stable solid compounds during coal gasification [36]. Thus, CGCS has great application potential in UHPC as a fine aggregate substitute.

**Figure 1:**
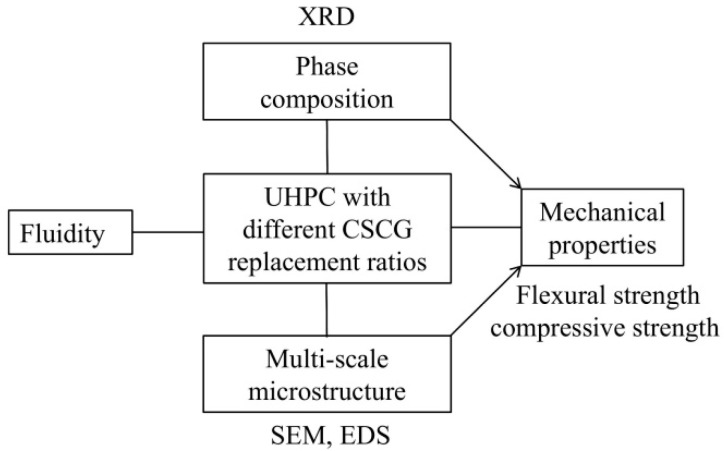
Flow chart of the research process.

This study investigated the application of CGCS in UHPC as an RS substitute. Figure 1 shows the flow chart of the research process. The fluidity and mechanical properties of specimens with different replacement ratios of CGCS were examined. The phase composition analysis was performed using X-ray diffraction (XRD) to examine the effect of the CGCS replacement ratio on the hydration products of specimens. The effect of CGCS replacement ratio on the mechanical properties was investigated through microstructure analysis, especially for interface transition zones (ITZs). Considering that the microstructures of specimens with CGCS were highly complex, multi-scale microstructure images with different replacement ratios of CGCS were obtained using scanning electron microscopy (SEM) and X-ray energy-dispersive spectroscopy (EDS), which were transformed from the micron- to the millimetre-scale to be more representative [37]. The findings of this study provide insights for developing new and clean industrial technologies for the use of CGCS and offer theoretical basis for the research of fine aggregate replacement in UHPC, which is essential for the low-carbon and green development of coal chemical engineering and construction industry in the middle–upper Yellow River basin and similar regions around the world.

## 2. Materials and Methods

### 2.1. Raw Materials

**Figure 2:**
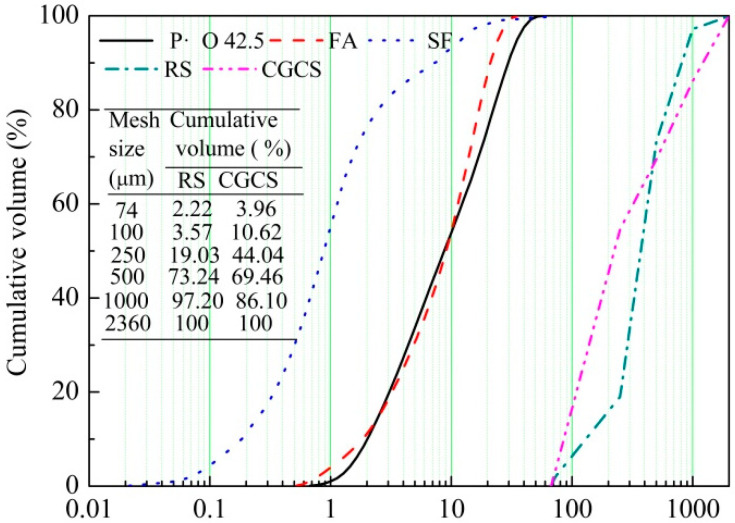
Particle size distribution of the raw materials.

RS, CGCS, ordinary Portland cement (P·O 42.5), fly ash (FA), and silica fume (SF) were the main raw materials used in the study. CGCS was collected from a coal chemical company in Yulin City for RS substitution. P·O 42.5 was produced under the Chinese standard [38]. FA and SF were added as supplementary cementitious materials. The mean particle size (D_50_) of P·O 42.5, FA, and SF was 10.3 μm, 9.9 μm, and 0.2 μm, respectively, as determined using a laser particle size analyser (MAZ 3000, London, England). Straight copper-coated steel fibres (length = 10 mm, diameter = 0.02 mm, slenderness = 500, density = 7850 kg·m^−^^3^, and tensile strength > 3000 MPa) were used to increase the flexural strength. A poly-carboxylic ether super-plasticiser (SP) with 55% solid content was used. The chemical compositions of raw materials determined using X-ray fluorescence (XRF, Panalytical, Amsterdam, The Netherlands) are listed in Table 1. The physical properties of raw materials are listed in Table 2, and their size distribution results are shown in Figure 2.

**Figure 3:**
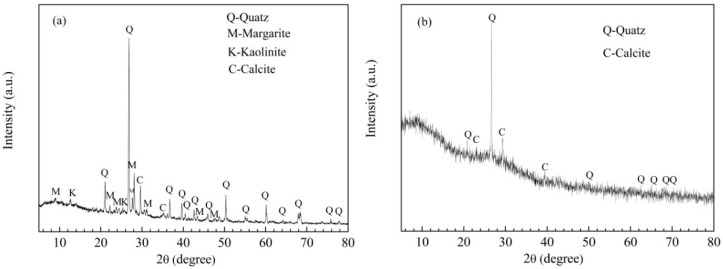
XRD patterns of fine aggregates: (**a**) RS, (**b**) CGCS (Quatz-SiO_2_, Margarite (CaAl_2_Si_2_Al_2_O_10_(OH)_2_), Calcite (CaCO_3_), Paraspurrite (Ca_5_(SiO_4_)_2_CO_3_)).

Phase compositions of fine aggregates were examined using XRD (shown in Figure 3). RS mainly comprised crystalline phases, including quartz (SiO_2_), margarite (CaAl_2_Si_2_Al_2_O_10_(OH)_2_), kaolinite (Al_2_(Si_2_O_5_)(OH)_4_), and calcite (CaCO_3_). CGCS exhibited abundant amorphous phases, such as glass phase, amorphous carbon phase [35], as well as quartz and calcite phases.

### 2.2. Mix Design

**Figure 4:**
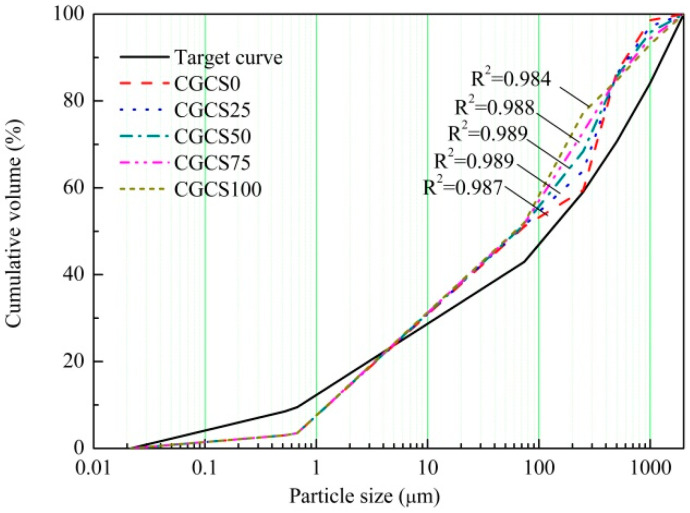
Particle size distribution of dry mixtures, R^2^ = correlation coefficient.

The designed mix proportions in this study are listed in Table 3. The mixture with RS as the fine aggregate was labelled CGCS0, and mixtures with CGCS replacement ratios of 25%, 50%, 75%, and 100% were labelled as CGCS25, CGCS50, CGCS75, and CGCS100, respectively. The CGCS replacement ratios were calculated by the weight fraction rather than the volume fraction as the apparent difference in density between RS and CGCS. To ensure strength when reducing costs and to further increase UHPC applications, a moderate proportion of P·O 42.5 cement was designed (495 kg·m^−3^, 23% in mass). The application of FA reduced the cost and improved the mechanical properties of the specimens. The proportions of FA and SF were determined according to the results of preliminary experiments. The modified Andreasen and Andersen model [38] was employed to evaluate the packing density of dry mixtures. As shown in Figure 4, the correlation coefficient (R^2^) between each mixture curve and the target curve was calculated, which was 0.987, 0.989, 0.988, 0.984 and 0.987 of CGCS0, CGCS25, CGCS50, CGCS75 and CGCS100, respectively, indicating that the obtained five mixtures are desirable. The detailed design process was adopted from a previous study [39].

Steel fibres were added in 1.9% volume fraction. SP and water were premixed in a ratio of 1:6.7 in mass. Their total proportions were controlled according to the fluidity of fresh mixtures. SP^S^ represents the mass of solid SP; water proportion is the total water content; and the water–binder ratio (w/b) was calculated by dividing the total water content by the total amount of binder (995 kg·m^−3^).

### 2.3. Specimen Preparation

Concrete mixtures were prepared in a mortar mixer by using well-established procedures described in previous studies [28,30]. First, all dry mixtures including RS, CGS, P·O 42.5, FA, and SF were stirred for 3 min at 140 rpm. Then, the mixtures were mixed at a higher speed of 280 rpm for 7 min, and 75% of a solution of SP and water was gradually added to the mixture. The remaining 25% solution was added drop-wise when the fluidity of the mixture had nearly approached 180 mm. Finally, steel fibres were added slowly and stirred at 140 rpm until homogeneous mixtures were obtained.

Fresh mixtures were poured into plastic moulds (dimensions: 40 mm × 40 mm × 160 mm) and vibrated for 2 min to remove bubbles formed in the mixing process. Then, the specimens were stored in a curing room at 20 ± 1 °C and 95% relative humidity (RH) for 48 h and covered with plastic sheets to avoid surface moisture loss. After de-moulding, the specimens were steam cured at 95 ± 1 °C and 95% RH for 24 h to promote the pozzolanic activity of CGCS [21]. Thereafter, the specimens were stored at 20 ± 1 °C and 95% RH until the pre-determined testing times (3, 7, 28, and 56 days). Twelve specimens were made for each series of concrete mixtures.

### 2.4. Test Methods

#### 2.4.1. Fluidity Test

To evaluate the workability of fresh mixtures, fluidity tests were carried out in accordance with the American Society for Testing Materials C230/C230M-14 standard [40]. Fresh mixtures to be tested included steel fibres.

#### 2.4.2. Mechanical Properties Tests

The flexural and compressive strengths of specimens on days 3, 7, 28, and 56 were measured using a TYE-300 universal testing machine (Changtaile, Changzhou, China) with a loading rate of 2 mm/min. The flexural strength was tested using a three-point method (span distance = 100 mm). The mean value of the flexural strengths of three specimens was reported as the flexural strength for each mixture. Thereafter, compressive strength tests were conducted on three broken specimens. The mean value of the compressive strengths of three specimens was reported as the compressive strength for each mixture. The water absorption and porosity of specimens on day 28 were measured in accordance with ASTM C1585-2013 [41].

#### 2.4.3. XRD and SEM Tests

X-ray Diffractomer (XRD, Empyrean, PANalytical B.V., Amsterdam, The Netherlands) at a scan rate (2θ) of 5°/min in the range 5–80° was used to examine the hydration products of specimens with different CGCS replacement ratios on days 7 and 28. The target material was copper target with a characteristic ray wavelength of 0.15406 nm. Scanning electron microscopy (SEM, VEGA II-XMU, TESCEN, Prague, Czech Republic) was used to examine the effect of the CGCS replacement ratio on the mechanical properties of specimens along with EDS.

Four micrometre-scale SEM images were seamlessly attached to achieve millimetre-scale SEM images to perform multi-scale microstructure analyses. Raw materials and cement hydration products were identified using EDS analysis. Pore structure and quantitative results of unreacted cement were studied using the millimetre-scale SEM images. The morphology of cement hydration products and their interfacial bonding with RS and CGCS in the ITZs were investigated using the micrometre-scale SEM images.

## 3. Results and Discussion

### 3.1. Fluidity

Many studies on concrete have focused on the relationship between fluidity and fine aggregate replacement ratio for a constant value of w/b [29,30,41]. These studies have shown that the mechanical properties and durability of UHPC can be improved by decreasing the w/b value. In this study, it was more meaningful to investigate the lowest w/b at different CGCS replacement ratios in the fresh mixtures to meet the minimum workability and provide a theoretical basis for engineering applications of UHPC with CGCS. The minimum fluidity of fresh mixtures was set to 180 mm in this study [42]. The variation in w/b with CGCS replacement ratios is shown in Table 3. When the fluidity was 180 mm, the w/b continuously increased from 0.22 to 0.37 with an increase in the CGCS replacement ratio.

**Figure 5:**
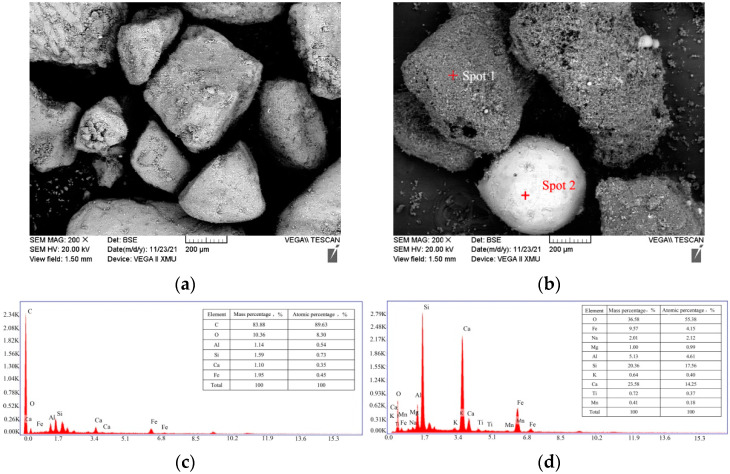
Microstructure of the SEM-EDS of fine aggregates: (**a**) SEM photo of RS, (**b**) SEM photo of CGCS, (**c**) EDS spectra of spot 1, (**d**) EDS spectra of spot 2.

The fluidity of UHPC is dominated by the particle packing density and the surface property of particles [43]. The R^2^ of dry mixtures varied slightly, as shown in Figure 4; the microstructures of RS and CGCS determined by SEM-EDS are shown in Figure 5. There is no obvious pore structure on the smooth surface of polygonal RS, and two characteristic morphologies can be observed in CGCS. Spot 1 shows that the irregular particles contain a considerable amount of carbon, which is unburned carbon [13]. The surface of unburned carbon is porous and rough. Several spherical beads with different particle sizes are also observed, whose surfaces are smooth and dense. During the gasification process, the minerals in the coal melt at a high temperature, generating many spherical particles after cooling under the effect of the surface energy [44]. The EDS results of spot 2 show that the spherical beads mainly comprised silicon, calcium, iron, aluminium, and oxygen. Quartz and calcite crystallise phases were excluded according to the proportion of atoms, and thus, the spherical beads comprised an amorphous glass phase.

The spherical glass beads with smooth surfaces were beneficial to reduce w/b by reducing the friction with cement slurry, and as a result, the dominant factor was the surface property of carbon particles such as roughness, water absorption, etc. At low w/b, the carbon particles were hydrophobic, and as a result, the cement slurry was dispersed around the surfaces of carbon particles and could not wet them. With increasing w/b, water entered the carbon particles via capillary action through open pores on the surfaces. The carbon particles were wetted and surrounded by the cement slurry. However, this process slightly changed the stiffness of the cement slurry because of partial water loss, and thus there was considerable internal friction resistance between fine aggregates and the fluidity was limited. The w/b increased until a cement slurry layer with adequate thickness was formed, and the fresh mixtures could obtain the desired fluidity. The hydrophobicity and high water absorption owing to the porous structure of CGCS were the main reasons for the increase in the w/b. In addition, fine CGCS particles absorbed more water than RS. Thus, the range of w/b was much higher than other studies on UHPC (0.18–0.22) [24,29,30].

### 3.2. Mechanical Properties

**Figure 6:**
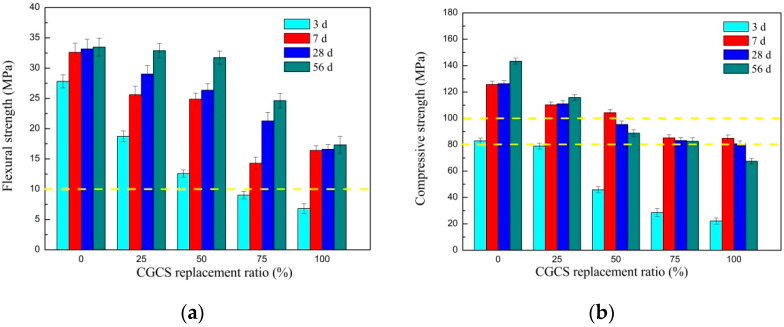
The mechanical strength of hardened specimens with different CGCS replacement ratios on day 3, 7, 28 and 56: (**a**) flexural strength, and (**b**) compressive strength.

The flexural and compressive strengths of specimens with different CGCS replacement ratios on days 3, 7, 28, and 56 are shown in Figure 6. The strength decreased with the increase in the CGCS replacement ratios, which aided the variation trend of porosity. After curing for 28 days, for CGCS replacement ratios of 25%, 50%, 75%, and 100%, the flexural and compressive strengths decreased by 13.2%, 21.2%, 36.4%, and 50.4% and 11.9%, 24.3%, 33.9%, and 36.0%, respectively. The degradation of strength may be attributed to poorer microstructure of mixtures with increasing CGCS replacement ratios, such as higher proportion of porous carbon particles, weaker bond strength between cement slurry and aggregates, which is discussed in detail in later sections based on the multi-scale microstructure analysis.

According to T/CES 10107-2020 [45], for CGCS replacement ratios of 0–100%, the flexural strengths of all the specimens were >10 MPa, and these specimens satisfied the flexural strength requirement of the structural UHPC; whereas, only specimens with CGCS replacement ratios of ≤25% met the non-structural UHPC grade with compressive strengths of >100 MPa. The compressive strength of specimens with CGCS replacement ratios of 25–100% was >80 MPa, and these specimens satisfied the compressive strength requirement of C80 HPC. However, the mechanical properties of specimens in this work were much lower than that of UHPC containing gold mine tailings [24], waste cathode ray tube funnel glass [29], and waste glass sand [30] as substitutions of fine aggregates. The relatively low cement proportion (495 kg·m^−3^, 23% in mass) and relatively high water absorption of CGCS were the main reasons for that.

With an increase in the curing age, the flexural strengths of specimens increased with increasing CGCS replacement ratios. Similar results were observed for compressive strengths when the CGCS replacement ratio was ≤25%. However, for CGCS replacement ratios of >25%, the compressive strength decreased after 28 days, which can be unfavourable during long-term usage. Mo et al. [46] investigated the mechanical performance of UHPC matrixes containing meta-kaolin (MK) under various steam curing conditions. Similar with CGCS, MK processes rich pore structure and high pozzolanic reactivity [46]. When the MK level was higher than 15%, steam curing at 90 °C for 24 h could remarkably enhance the compressive strength for UHPC matrix at early age. However, the compressive strength decreased considerably after the end of steam curing with age, which is in accordance with the present study. It was believed that the main reason was heterogeneous microstructure caused by high water absorption of MK grains [46]. The crack development with curing age induced by early age might be another reason for the decreasing trend of the compressive strength [47].

**Figure 7:**
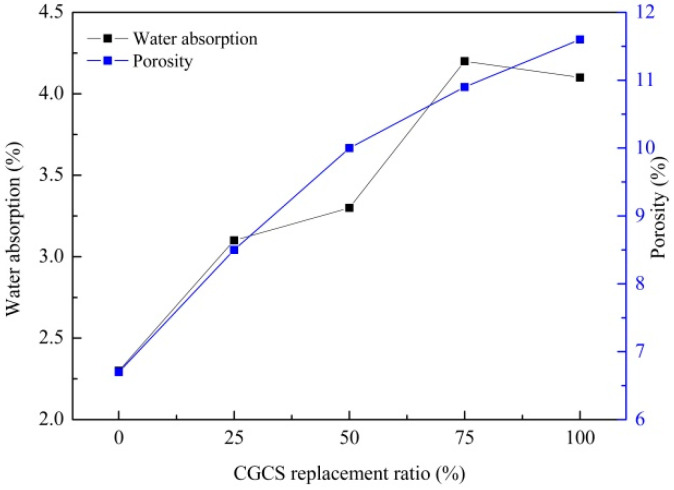
Water absorption and porosity of specimens with different CGCS replacement ratios.

Water absorption and porosity directly influence the durability of UHPC. Figure 7 shows their values with different CGCS replacement ratios at 28 days, and they increased with increasing CGCS replacement ratios. The water absorption and porosity of CGCS100 were 11.6% and 4.1%, respectively, which are approximately twice those of CGCS0. The internal porosity of CGCS and external hole caused by increasing w/b were the major influencing factors.

### 3.3. Hydration Products

**Figure 8:**
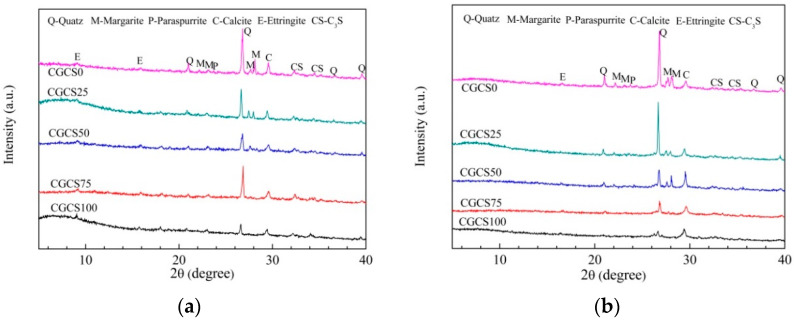
XRD patterns of specimens with different CGCS replacement ratios: (**a**) 7 days, (**b**) 28 days.

Figure 8 shows the XRD patterns of specimens with different CGCS replacement ratios on day 7 and 28. The diffraction peaks show three types of particles, namely, fine aggregates, hydration products, and unreacted cement.

The phases of fine aggregates mainly included quartz, margarite, kaolinite, and calcite. On day 7 and with increasing CGCS replacement ratios, peaks of quartz, margarite and kaolinite were weakened, which is closely related to the phase composition of RS and CGCS. Similar XRD patterns were observed on day 28.

On days 7 and 28, the primary hydration products were ettringite (AFt). Crystallisation peaks of calcium silicate hydrate (C–S–H) were not observed because they typically exist as gel in concretes [48]. On day 7, with increasing CGCS replacement ratios, the peaks of AFt were stronger. High-temperature steam curing led to the high pozzolanic activity of the amorphous glass phase in CGCS, and it reacted with calcium hydroxide in the cement slurry to form AFt and C–S–H [20]. Thus, AFt increased with increasing CGCS replacement ratio and that improved the early strength. This also explained the high early strength of specimens with different CGCS replacement ratios on day 7 (Figure 6). On day 28, XRD peaks of AFt were relatively weak, and these converted to monosulfoaluminate hydrate (AFm) with increasing hydration age. However, because of the low amount of AFm, corresponding peaks were not observed. As shown in Table 1, the proportions of SO_3_ in CGCS were higher than those of RS. At the later hydration stage, excess SO_3_ reacted with AFt to form AFm, resulting in 1.5 times volume expansion [49]. Thus, the compressive strengths of specimens with CGCS replacement ratios >25% decreased after 7 days.

Unreacted tricalcium silicate (C_3_S) was observed in the specimens aged 7 and 28 days. Peaks of C_3_S on day 28 were weaker than those on day 7, indicating more complete hydration reactions.

### 3.4. Microstructure Analysis

#### 3.4.1. Internal Hydration Effect of CGCS

**Figure 9:**
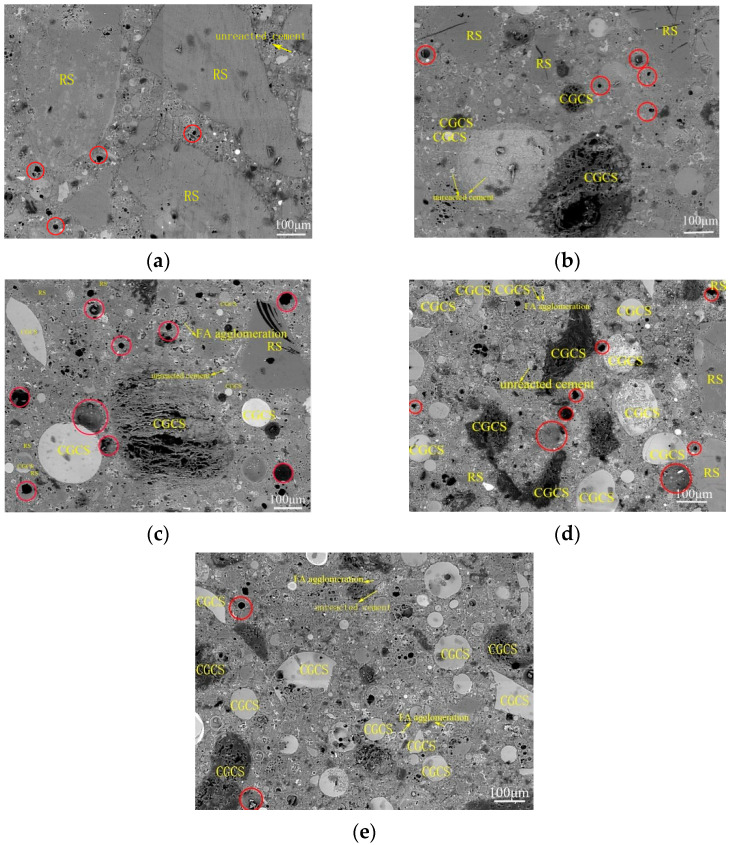
Millimetre-scale back scattering electron microscope (BSE) images of specimens with different CGCS replacement ratios on day 28: (**a**) 0%, (**b**) 25%, (**c**) 50%, (**d**) 75%, (**e**) 100%. (The areas circled in red represent pores with a size > 10 μm).

Figure 9 shows millimetre-scale back scattering electron microscopy (BSE) images of the specimens with different CGCS replacement ratios on day 28; these images mainly show the distribution of elements on specimen surfaces [28]. The brightest areas represent the existence of Ca or Fe, where the darkest areas indicate C abundance or pores. Thus, RS and CGCS can be distinguished from each other. However, distinguishing CGCS powder from FA or unreacted cement particles from hydration products is difficult.

**Figure 10:**
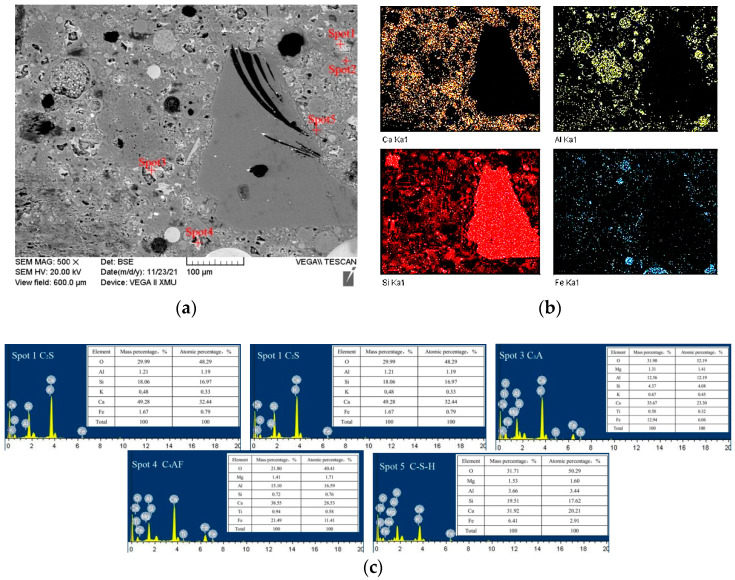
The distribution of raw materials and hydration products in a typical zone of CGCS50: (**a**) SEM photo, (**b**) Element mapping of Ca, Al, Si and Fe elements, (**c**) ESD spectra of spot 1–5, which represents for C2S, C3S, C3A, C4AF, C–S–H, respectively.

Figure 10 shows the distribution of raw materials and cement hydration products of a typical zone in CGCS50, which were identified with the EDS mapping results (Figure 10b) assisted with point analysis (Figure 9c). Irregularly shaped aggregates rich in Si are RS, where in CGCS the solid spherical aggregates rich in Ca are glass beads. The porous dark-coloured particles are carbon present in CGCS. The hollow spherical particles rich in aluminium are FA. The solid spherical particles rich in aluminium are FA, or those rich in Ca element are CGCS powders. The unreacted cement particles are mainly dicalcium silicate (C_2_S), C_3_S, tricalcium aluminate (C_3_A), and tetracalcium ferroaluminate (C_4_AF), which tend to agglomerate in irregular bands. Hydration products are dispersed around the fine aggregates and mainly include C–S–H. Spherical glass beads and porous dark carbon particles increased with the increase in the CGCS replacement ratio.

**Figure 11:**
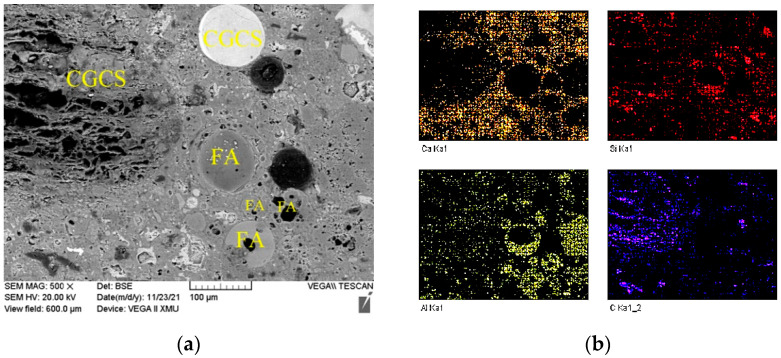
Microstructure of a typical zone in mixture CGCS50: (**a**) SEM photo, (**b**) Element mapping of Ca, Al, Si and C elements.

Although porous carbon particles in CGCS negatively affected the fluidity and mechanical properties of specimens in many ways, it showed strong interfacial bonding with cement slurry (Figure 9). Few cracks or unreacted cement particles were observed around porous carbon particles in all the specimens, except in CGCS100. Figure 11 shows the microstructure and EDS mapping results of another typical zone in CGCS50. The distribution of Ca element was mainly observed to analyse the amount and distribution of hydration products around different raw materials. It can be seen that a considerable number and continuously distributed hydration products are formed around glass beads and FA particles owing to their high pozzolanic activity. The same was observed around porous carbon particles. These results could be explained by the internal hydration effect [14]. The pore carbon particles absorbed water during mixing and released it after setting. When the amount of water was inadequate in concrete, the porous carbon particles released water and ensured the complete chemical reaction with the surrounding cement particles. The internal hydration effect of CGCS promoted the hydration process to a certain extent and helped improve the performance of the interface transition zone and mechanical properties.

#### 3.4.2. Pore Structure

The pore structure is an important factor affecting the mechanical properties and durability of concrete. Pores with different shapes and sizes of CGCS0 and other materials are shown in Figure 9. In particular, pores with a size > 10 μm (circled by red) were observed with the increase in the CGCS replacement ratio. The large hole in CGCS0 may indicate the packing hole or residual air bubble. With the increase in the CGCS replacement ratio, additional large spherical holes were observed, and many pores were introduced by CGCS and FA. The w/b increased with the increase in the CGCS replacement ratio due to the high water absorption of CGCS. Excess water available was neither in close contact with the aggregates nor could it rapidly participate in the hydration process; thus, this isolated excess water led to the formation of large spherical holes. The large holes were mainly distributed in the cement slurry rather than around large aggregates, which reduced the strength of the cement slurry and weakened the mechanical properties of concrete. On the other hand, the pore in carbon particles led to a reduction in the pressure area; thus, the flexural and compressive strengths decreased with the increase in the CGCS replacement ratio on days 3, 7, 28, and 56 (Figure 5). To improve the mechanical properties, it is necessary to modify the surface of CGCS to reduce the w/b and increase the strength of porous carbon particles.

Glass bead-like CGCS particles have a unique spherical structure and dense smooth surface. They are uniformly dispersed in the cement slurry (Figure 9), which led to a decrease in the friction between fine aggregates and cement slurry [48] and filling of gaps in the slurry, thus further improving the mechanical properties. Therefore, the compressive strength of specimens with a CGCS replacement ratio of 100% on day 28 was >80 MPa.

#### 3.4.3. Unreacted Cement

**Figure 12:**
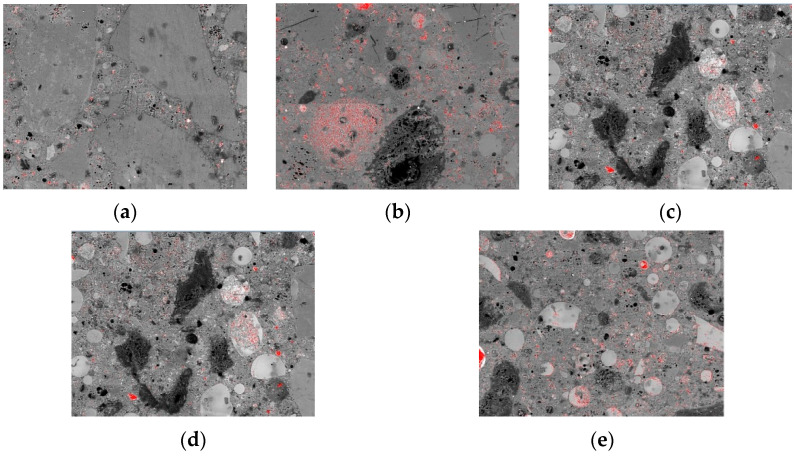
The unreacted cement spots distributions of specimens with different CGCS replacement ratios on day 28 (red spots represent for unreacted cement): (**a**) 0%, (**b**) 25%, (**c**) 50%, (**d**) 75%, (**e**) 100%.

Some unreacted cement particles are seen in Figure 9. As millimetre-scale images are highly representative, the statistical analysis of unreacted cement amount based on these images is effective in evaluating the hydration degree of specimens with different CGCS replacement ratios on day 28. The quantitative results of unreacted cement were considered to represent the hydration degree of specimens. The unreacted cement spots were extracted, and their area was determined by using image pro plus software (Figure 12). The area of unreacted cement of specimens with the CGCS replacement ratios of 0%, 25%, 50%, 75%, and 100% was 65, 158, 2189, 4086, and 4728 pixels, respectively, which increased with the increase in the CGCS replacement ratio, indicating that CGCS hindered cement hydration. The hydration reaction between fine aggregates and cementitious materials was inhibited by the high content of residual carbon in the CGCS, which hindered the growth and connection of hydration products and weakened the mechanical properties of mixtures. With the increase in CGCS replacement, the agglomeration phenomenon of FA became increasingly obvious (Figure 9), thereby affecting the secondary hydration and the strength of concrete [50,51].

#### 3.4.4. ITZ Microstructure

**Figure 13:**
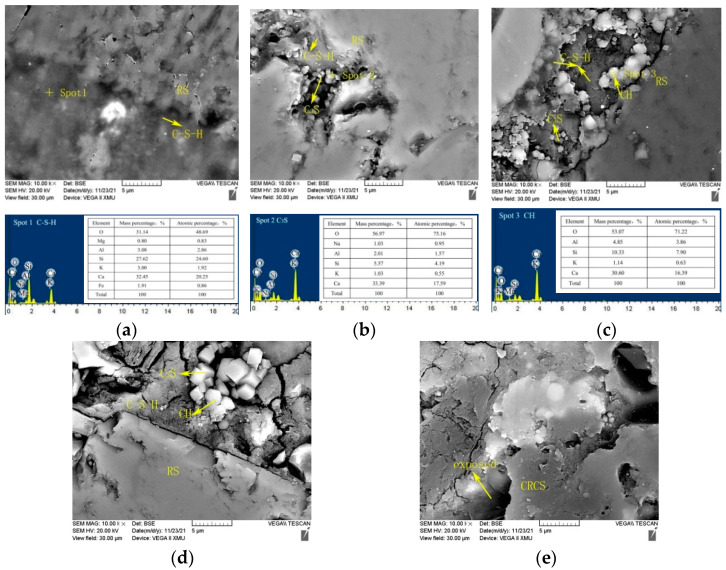
Microstructure of interface transition zone around the large fine aggregates of specimens with different CGCS replacement ratios on day 28: (**a**) 0%, (**b**) 25%, (**c**) 50%, (**d**) 75%, (**e**) 100%.

ITZ is the weakest structural region in concrete [48]. Figure 13 shows ITZ around the large fine aggregates of specimens with different CGCS replacement ratios on day 28. When the fine aggregates were RS (Figure 13a), RS was tightly wrapped with the cement slurry and no obvious cracks appeared around it. The massive gel around RS was C–S–H, which exhibited strong binding strength with the RS interface. Moreover, C–S–H was tightly connected to the RS aggregates in the specimen CGCS25 (Figure 13b); however, a small amount of cubic unreacted C_3_S appeared. For CGCS50 (Figure 13c), long cracks were observed around RS, and a large number of lamellar calcium dihydroxide (CH) grew directionally on the interface and C–S–H and cubic unreacted C_3_S were observed. This loose structure was the typical characteristic of ITZ, which mainly contributes to reducing the mechanical strength of CGCS50. For CGCS75 (Figure 13d), many cracks and unreacted C_3_S were observed around the RS aggregate. When all the fine aggregates were of CGCS (Figure 13e), the surface area of CGCS increased due to low density; thus, partial CGCS was exposed, causing a sharp decrease in mechanical strength. The increase in the C_3_S quantity (Figure 13) also confirmed that CGCS hindered the reaction in the later stage of hydration.

Based on the multi-scale microstructure analysis, the effect of the CGCS replacement ratio on the mechanical properties of mixtures was determined here. The positive effects of the CGCS replacement ratio on the mechanical properties can be summarised as follows: (1) glass bead-like particles in the CGCS reduced the friction between fine aggregates and cement slurry and filled gaps in the slurry; (2) due to the pozzolanic activity of glass bead-like particles in CGCS, the AFt amount increased with the increase in the CGCS replacement ratio on day 7; (3) porous carbon particles in the CGCS showed strong interfacial bonding with cement slurry due to internal hydration.

However, the flexural and compressive strengths decreased with the increase in CGCS replacement ratio. On the one hand, large pores formed at the higher values of w/b caused by porous carbon particles in CGCS, which considerably reduced the strength of the cement slurry. On the other hand, the residual carbon particles in CGCS hindered the hydration process in the later stage. This was confirmed with the quantitative results of unreacted cement and FA agglomeration based on the millimetre-scale SEM images, and the increase in cubic unreacted C_3_S as can be seen in the micrometre-scale SEM images. In addition, ITZ structures formed when the CGCS replacement ratio was up to 50%, which weakened the bond strength between the aggregates and cement slurry. It is necessary to carry out surface modification of CGCS to reduce the water absorption and improve the aggregate strength in the following work, and also to increase the mechanical properties of UHPC with CGCS as river sand replacement.

## 4. Conclusions

In this study, the application of CGCS in UHPC as an RS substitute was investigated. The fluidity, hydration products and mechanical properties of specimens with different CGCS replacement ratios were studied. The effects of different CGCS replacement ratios on mechanical properties were examined based on multi-scale microstructures analysis through XRD, SEM, and EDS. The main results are as follows:

(1) CGCS has a potential for application in UHPC as RS replacement. The mechanical properties of specimens with CGCS replacement ratios of ≤25% satisfy the strength requirement of non-structural UHPC. The flexure and compressive strengths of specimens containing 25% CGCS were 29 and 111 MPa, respectively;

(2) The w/b, water absorption and porosity increased with the increase in the CGCS replacement ratio. The increase in the w/b was mainly attributed to the hydrophobicity and high water absorption of porous carbon particles in CGCS, which further resulted in the increase in water absorption and porosity;

(3) With increasing CGCS replacement ratios, the flexural and compressive strengths of specimens decreased on days 3, 7, 28, and 56. The compressive strength increased with the curing age when the CGCS replacement ratio was ≤25%. However, it decreased when the CGCS replacement ratio was >25%;

(4) The AFt amount increased with the increase in the CGCS replacement ratio on day 7 owing to the pozzolanic activity of glass bead-like particles in CGCS. However, on day 28, XRD peaks of AFt were relatively weak, and these might convert to AFm, which led to decreasing compressive strength with increasing hydration age;

(5) Porous carbon particles in CGCS showed strong interfacial bonding with cement slurry due to internal hydration. However, the pore quantity, the unreacted cement and the agglomeration of FA increased with the increase in the CGCS replacement ratio. In addition, ITZ structures formed when the CGCS replacement ratio was up to 50%. CGCS hindered the hydration process in the later stage. Therefore, the flexural and compressive strengths decreased with the increase in CGCS replacement ratio.

Further research should focus on the surface modification of CGCS for enhanced mechanical properties, the possibility of alkali–silica reaction of CGCS, the durability of specimens with different CGCS replacement ratios, and the systematic effect mechanism of CGCS replacement on the hydration behaviour. In our future studies, we aim to explore the large-scale industrialisation of CGCS and its considerable environmental and economic significance.

## Figures and Tables

**Table 1:** Chemical composition of fine aggregates and powders by mass fraction (%).

Material	SiO_2_	Al_2_O_3_	Fe_2_O_3_	CaO	MgO	Na_2_O	K_2_O	TiO_2_	SO_3_	P_2_O_5_	Loss
RS	68.46	15.65	5.05	3.69	0.05	1.75	2.42	0.86	0.22	0.30	1.85
CGCS	41.94	15.46	17.70	12.61	1.68	1.71	1.36	0.71	2.48	0.21	9.16
P·O 42.5	23.04	4.50	4.78	64.81	2.02	0.31	0.22	-	0.636	-	2.34
FA	42.43	21.83	12.81	15.12	2.12	2.04	1.02	-	-	-	0.42
SF	96.78	0.78	0.56	0.64	0.73	0.78	0.67	-	-	-	2.23

**Table 2:** Physical properties of fine aggregates.

Material	Maximum Particle Size (μm)	Apparent Density (kg·m^−3^)	24 h Water Absorption (%)
RS	2360	2480	0.56
CGCS	2360	1630	4.0

**Table 3:** Mix proportions design (kg·m^−3^).

Mixture	RS	CGS	P·O 42.5	FA	SF	Steel Fibre	SP^S^	Water	w/b
CGCS0	995	0	495	335	165	150	17	222	0.22
CGCS25	746	249	495	335	165	150	17	231	0.23
CGCS50	497	498	495	335	165	150	21	277	0.28
CGCS75	248	747	495	335	165	150	23	304	0.31
CGCS100	0	995	495	335	165	150	28	369	0.37

## Data Availability

Not applicable.
